# Evaluation of antimicrobial potential and cytotoxic of *Pouteria venosa *species

**DOI:** 10.1186/1753-6561-8-S4-P6

**Published:** 2014-10-01

**Authors:** Raíssa Fernanda Evangelista Pires dos Santos, Isabelle Souza de Mélo Silva Silva, Eleci Adriano Hendges, Andressa Letícia Lopes da Silva, Andriele Mendonça Barbosa, Klebson Silva Santos, Renata Rego de Souza, Thais Honório Lins, Regina Célia Santos Sales Verissimo, Francine Ferreira Padilha, Eliane Aparecida Campesatto, Maria Lysete de Assis Bastos

**Affiliations:** 1 Laboratório de Pesquisa em Tratamento de Feridas, Universidade Federal de Alagoas, Campus A. C. Simões, Av. Lourival Melo Mota, s/n, Cidade Universitária, Maceió, Alagoas, CEP 57072-900, Brasil; 2 Instituto de Tecnologia e Pesquisa, Universidade Tiradentes - Av. Murilo Dantas, 300, Farolândia, Aracaju, Sergipe, CEP 49032-490, Brasil; 3 Empresa Brasileira de Pesquisa Agropecuária, Av. Beira Mar, 3250, Jardins, Aracaju, Sergipe, CEP 49025-040, Brasil; 4 Laboratório de Farmacologia e Imunidade, Universidade Federal de Alagoas, Campus A. C. Simões, Av. Lourival Melo Mota, s/n, Cidade Universitária - Maceió, Alagoas, CEP 57072-900, Brasil

## Background

The use and research of medicinal plants in Brazil have as allies the great plant diversity and low cost associated with treatment [1]. The popular usage relates to the species of the family Sapotaceae which reports antibacterial, antifungal, antiviral, antitumor, analgesic, antipyretic, anti-inflammatory, and others [2]. It was shown that the species of genus Pouteria, belonging to the Sapotaceae family, have proven biological activities, it is cited *Pouteria caimito *with antioxidant and photoprotection activity against UVA and UVB; Pouteria ramiflora with antinociceptive, anti-inflammatory, antioxidant, photoprotection activity (against UVA and UVB), antimicrobial activity and toxicity against larvae of Brine Shrimp, and *Pouteria torta *with antimicrobial and antifungal activity [3]. Phytochemical studies performed with species of Sapotaceae have revealed the presence of alkaloids, flavonoids, terpenoids, benzenoids, phenylpropanoids and Lapachol, responsible for large spectrum of biological activities [4]. Since were reported promising biological activities of the genus Pouteria, belonging to the Sapotaceae family, and their constituents, and considering that the species of *Pouteria venosa*, known as "tuturubá, leiteiro, Bapeba, Sapota black", has not yet defined its antimicrobial activity, aimed to evaluate the antimicrobial and cytotoxic potential in view of the bacterial and fungal infections control.

## Methods

*In vitro *experimental study, conducted at the Research Laboratory of Wound Care at Federal University of Alagoas. It was evaluated four fractions and crude extract parts of the species *Pouteria venosa *named as samples A, B, C, D and E. Antimicrobial activity was determined by microbial sensitivity tests, the method of disk diffusion and broth microdilution method for determination of minimum inhibitory concentration (MIC). It was used 15 strains of microorganisms, among them Gram-positive and Gram-negative bacteria and fungi like *Candida albicans, Saccharomyces cerevisiae *and *Aspergillus brasiliensis*. Distributed by American Type Cell Cellection. Was obtained to evaluate the cytotoxicity by means of Metiltetrazolium colorimetric method which investigated the cell viability front of the samples tested.

## Results and conclusions

The samples demonstrated antimicrobial activity in eight of the fifteen microorganisms evaluated in the disk diffusion test. Three of Gram-positive bacteria: *Staphylococcus aureus, Staphylococcus epidermidis, Streptococcus pneumoniae*, and five Gram-negative bacteria: *Pseudomonas aeruginosa, Shigella flexneri, Proteus mirabilis, Acinetobacter calcoaceticus *and *Enterobacter aerogenes*. Samples A, B and C showed high antibacterial potential front *S. aureus, S. epidermidis, S. pneumoniae *and *P. aeruginosa *(inhibition zones ≥14). The results obtained by determination of the MIC of these strains showed that the fraction of sample C was considered with better antimicrobial activity, inhibiting microbial growth at concentrations between 1000 and 250 μg mL-1. These findings corroborate with the literature, since the species *Pouteria torta, Pouteria pallida e Pouteria ramiflora*, also showed antimicrobial activity against these microorganisms [3,5]. The fungi evaluated were not sensitive to the *Pouteria venosa *samples. Sample C was considered non-toxic at the concentration of 200 μg mL-1, and is considered a promising route of pre-clinical in vivo. It emphasized the importance of the outcomes from the perspective of development and innovation of new therapeutic alternatives in infection control.
